# Esketamine Preserves Network Connectivity and Promotes Recovery in Consciousness Disorders

**DOI:** 10.1002/cns.70890

**Published:** 2026-05-13

**Authors:** Xuewei Qin, Xuanling Chen, Lan Yao, Bo Wang, Hongchuan Niu, Zhenhu Liang, Zhibin Zhao, Jian Wang, Jiapeng Huang, Xiangyang Guo, Xiaoli Li

**Affiliations:** ^1^ Department of Anesthesiology Peking University International Hospital Beijing China; ^2^ Department of Neurosurgery Peking University International Hospital Beijing China; ^3^ Institute of Electrical Engineering Yanshan University Qinhuangdao China; ^4^ Department of Anesthesiology and Perioperative Medicine University of Louisville Louisville Kentucky USA; ^5^ Department of Anesthesiology Peking University Third Hospital Beijing China; ^6^ State Key Laboratory of Cognitive Neuroscience and Learning, IDG/McGovern Institute for Brain Research Beijing Normal University Beijing China

**Keywords:** coma recovery scale–revised, disorders of consciousness, electroencephalography, esketamine, network connectivity, propofol

## Abstract

**Background:**

Disorders of consciousness (DoC) present significant therapeutic challenges with limited effective interventions. The choice of anesthetic agent during procedures such as spinal cord stimulation may influence long‐term neurological outcomes, yet comparative evidence is scarce. This prospective, non‐randomized, comparative effectiveness trial investigated the neurophysiological and clinical effects of esketamine versus propofol in patients with DoC.

**Methods:**

In this prospective, non‐randomized trial, 34 adult DoC patients undergoing spinal cord stimulator implantation were allocated to receive either esketamine (*n* = 17) or propofol (*n* = 17) as the primary anesthetic, based on clinical practice. Multimodal assessments included the Coma Recovery Scale–Revised (CRS‐R) at baseline and 3‐month follow‐up, along with high‐density electroencephalography (EEG) to analyze permutation entropy (PE) and weighted phase lag index (wPLI). Multivariable regression adjusted for key confounders.

**Results:**

Compared to propofol, esketamine was associated with significantly faster recovery of spontaneous respiration (12.02 ± 3.88 vs. 17.42 ± 4.62 min, *p* = 0.005) and reduced need for intraoperative vasopressors (17.65% vs. 52.94%, *p* = 0.034). EEG analysis revealed that esketamine better preserved brain electrical complexity (higher permutation entropy during maintenance, *p* < 0.001) and maintained higher functional connectivity, particularly in the gamma band (weighted phase lag index, *p* = 0.054).

**Conclusion:**

Esketamine demonstrates superior neurophysiological preservation over propofol in patients with disorders of consciousness, maintaining neural complexity and gamma‐band connectivity during anesthesia. These mechanisms are associated with accelerated respiratory recovery, hemodynamic stability, and significantly improved consciousness outcomes at 3 months, supporting its potential as a neuroprotective anesthetic in this population.

## Graphical abstract

Esketamine anesthesia promotes consciousness recovery in patients with disorders of consciousness by preserving brain network complexity and connectivity during surgery. Compared to propofol, esketamine facilitates superior neurophysiological preservation and shows a strong trend toward improved long‐term clinical outcomes.

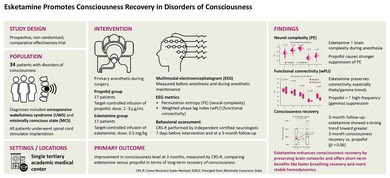

## 1 Introduction

Disorders of consciousness (DoC), encompassing conditions such as unresponsive wakefulness syndrome (UWS) and a minimally conscious state (MCS) [1, 2, 3], pose profound challenges in neurocritical care, with limited therapeutic options and unpredictable recovery trajectories [4, 5, 6, 7]. Novel interventions, such as spinal cord stimulation (SCS), hold promise in modulating neural circuits to promote arousal [8, 9]. However, the impact of general anesthesia on long‐term neurological outcomes remains largely overlooked. The anesthetic agent selected may constitute a critical, yet under‐investigated, determinant of recovery in this vulnerable population.

Propofol and esketamine exert their anesthetic effects through fundamentally opposing neurophysiological mechanisms. Propofol, a potent gamma‐aminobutyric acid (GABA) A receptor agonist, enhances inhibitory neurotransmission, widely suppressing cortical activity and markedly reducing high‐frequency oscillations [10, 11]. While effective in inducing unconsciousness, its profound cortical inhibition may inadvertently impede network reorganization, which is essential for the recovery of patients with DoC [12, 13, 14, 15, 16]. In contrast, esketamine, an N‐methyl‐D‐aspartate (NMDA) receptor antagonist, uses a disinhibitory mechanism [17, 18, 19]. Blocking NMDA receptors may mitigate excitotoxicity, promote neuroplasticity, and potentially preserve higher‐order network dynamics, even under anesthesia [20, 21, 22].

Nevertheless, robust evidence comparing the long‐term neurophysiological and clinical effects of esketamine and propofol in DoC remains scarce. We sought to pave the way for biomarker‐driven, individualized anesthetic strategies that offer a novel therapeutic avenue for DoC intervention. We hypothesized that esketamine, through its unique action as an NMDA receptor antagonist, would confer neuroprotective advantages over propofol by maintaining neural complexity and functional connectivity during anesthesia, thereby fostering both short‐term recovery and long‐term improvement in consciousness. We tested this hypothesis systematically by employing a multimodal assessment framework (standardized behavioral evaluation using the Coma Recovery Scale–Revised [CRS‐R] and high‐density electroencephalography [EEG]). This approach enabled us to delineate the dynamic neural mechanisms through which esketamine facilitates consciousness recovery, focusing on alterations in brain network oscillations and etiology‐specific response patterns.

## 2 Methods

### 2.1 Study Participants

The study cohort comprised 34 adult patients diagnosed with DoC who underwent spinal cord stimulator implantation at our neurosurgery department between February 2023 and January 2025.

Participants were eligible for inclusion if they met the following criteria: age 18–65 years; diagnosis of vegetative or MCS, a disease duration of at least 1 month, and an etiology including traumatic brain injury (TBI), hypoxic brain injury, or cerebrovascular accident; had not received sedative drugs for more than 72 h prior to the study; informed consent for participation in the study provided by their guardian.

Participants were excluded if they met any of the following conditions: concurrent primary neurological disorders, such as brain tumor, Parkinson's disease, or Alzheimer's disease; severe organic diseases, including heart disease of NYHA class III or higher, liver failure of Child–Pugh class C, or renal failure of CKD stage 4–5; history of anesthetic drug allergy; participation in other clinical trials within 30 days prior to enrollment; incomplete clinical data.

**FIGURE 1:**
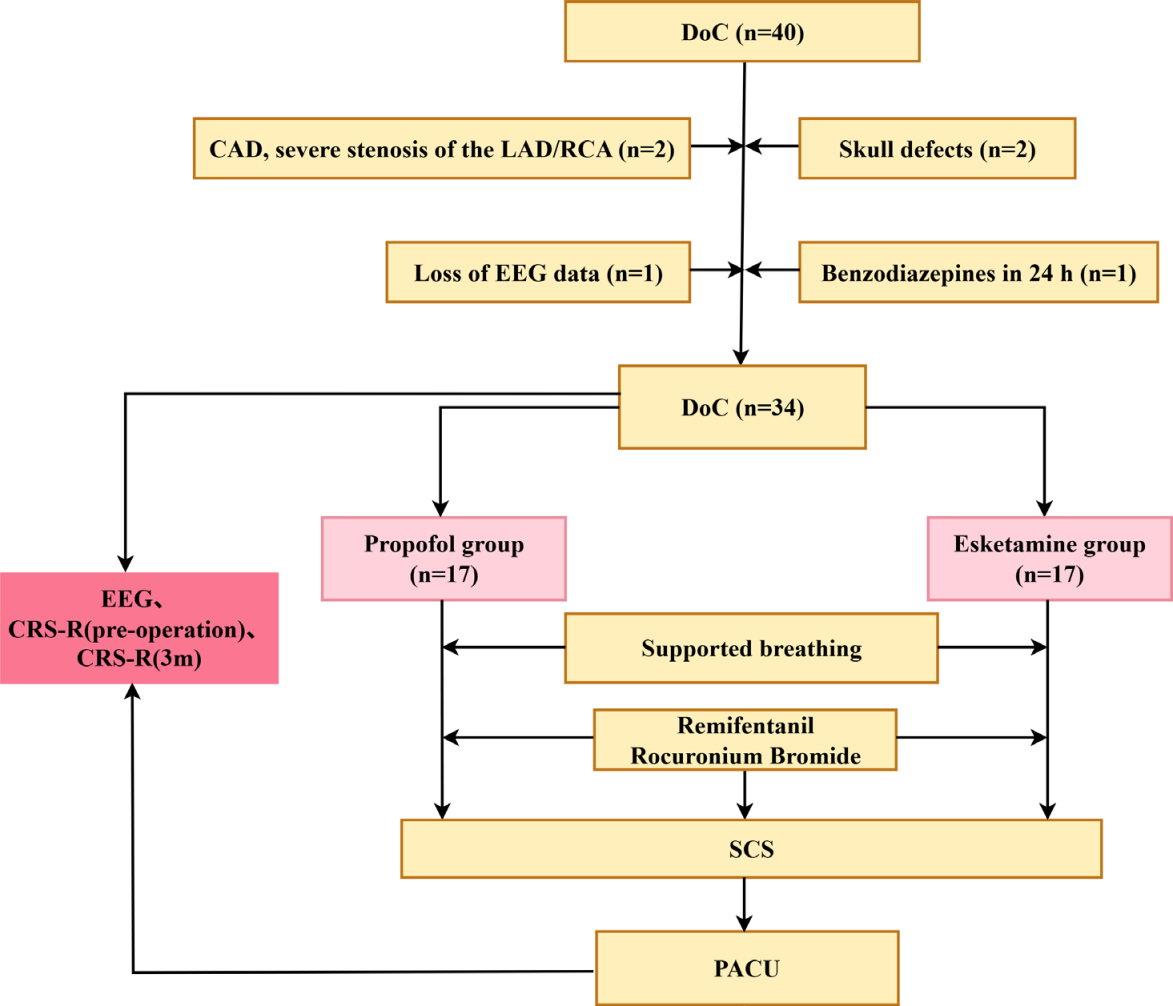
Flow diagram of the study. CRS‐R, Coma Recovery Scale–Revised; DoC, disorders of consciousness; EEG, electroencephalogram; PACU, post‐anesthesia care unit; SCS, spinal cord stimulation.

After rigorous screening, 34 eligible cases were enrolled and allocated to either the propofol or esketamine group (*n* = 17/group) (Figure 1).

### 2.2 Study Design and Group Allocation

This was a prospective, non‐randomized, comparative effectiveness trial. The choice of primary anesthetic agent (esketamine vs. propofol) was determined solely by the attending anesthesiologist based on standard clinical practice, individual patient characteristics (e.g., hemodynamic stability and anticipated pain), and institutional guidelines. The research team did not intervene in clinical decision‐making processes.

### 2.3 Baseline Assessment and Standardized Diagnostic Procedure

Prior to enrollment, all patients were comprehensively assessed at baseline to confirm their eligibility and establish their pre‐intervention status. In the general physiological assessments, vital signs were systematically monitored to ensure hemodynamic stability. Laboratory evaluations, including complete blood count, serum electrolyte panel, and coagulation profile, were mandated to fall within clinically acceptable parameters. To mitigate the confounding effect of prolonged sedation on consciousness evaluation, we required that all patients demonstrate a stable, spontaneous breathing pattern. Patients with tracheostomies had to have been successfully weaned from mechanical ventilation and to maintain stable spontaneous respiration for at least 72 h prior to the baseline assessment. UWS or MCS was defined strictly by established international diagnostic criteria, using the CRS‐R. Specifically, the classification of consciousness states—including UWS, MCS− (minimally conscious state without language‐related behavior), MCS+ (minimally conscious state with language‐related behavior), and EMCS (emergence from minimally conscious state)—was based on the operational definitions provided in the CRS‐R manual and aligned with internationally recognized diagnostic guidelines [23, 24, 25]. To ensure diagnostic precision and reduce the likelihood of misclassification, each patient underwent at least two CRS‐R evaluations performed by two independent certified neurologists on different days within the week pre‐intervention. A conclusive diagnosis was based on the highest level of responsiveness recorded in these evaluations. Diagnostic discrepancies between the assessors were resolved through discussion to consensus or consultation with a third senior neurologist.

### 2.4 Anesthesia Protocol Design and Perioperative Management

Upon admission to the operating room, a comprehensive multidimensional vital sign monitoring system was implemented for all patients, which included continuous monitoring of electrocardiogram, pulse oximetry (SpO_2_), and invasive radial artery pressure, with capabilities for monitoring circulatory dynamics and blood gas sampling, core body temperature, and bispectral index (BIS) targeted at 40–60. Anesthesia was induced by maintaining spontaneous respiration with a tracheal tube connected to a breathing circuit. Anesthesia was induced by target‐controlled infusion (TCI) of propofol at a concentration of 2–3 μg/mL (propofol group) or esketamine at a dose of 0.5 mg/kg (esketamine group). Upon confirmation of consciousness suppression, indicated by absence of the eyelash reflex, sufentanil was administered sequentially at a dose of 0.3 μg/kg for analgesia, followed by rocuronium at 0.5 mg/kg for skeletal muscle relaxation. Mechanical ventilation was commenced upon suppression of spontaneous breathing, aiming to maintain an end‐tidal carbon dioxide pressure within 35–45 mmHg.

During anesthesia maintenance, the propofol group received a continuous propofol infusion at a dosage of 1–1.5 mg/kg/h, combined with remifentanil at 0.15 μg/kg/min to sustain the targeted anesthetic depth. The esketamine group received a continuous esketamine infusion at 1 mg/kg/h combined with an equivalent remifentanil dose.

### 2.5 Definition of Respiratory Recovery Endpoint

The primary clinical endpoint, time to spontaneous respiration recovery, was rigorously defined. It was quantified as the duration (in minutes) from the termination of all study‐related anesthetic and analgesic infusions (specifically, propofol/esketamine and remifentanil) to the point at which the patient consistently fulfilled predefined criteria indicative of adequate respiratory function. These criteria included a spontaneous respiratory rate between 8 and 20 breaths per minute, the SpO_2_ of at least 92% (or at the preoperative baseline), and an end‐tidal CO_2_ (EtCO_2_) level between 35 and 45 mmHg, all maintained for a minimum of 10 consecutive minutes. This evaluation was performed in the post‐anesthesia care unit by an anesthesiologist who was blinded to the group allocation.

### 2.6 EEG Data Collection and Preprocessing

High‐density EEG was recorded using a 64‐channel cap arranged according to the international 10–20 system (WaveGuard EEG cap, ANT Neuro, The Netherlands). Data were acquired at a sampling rate of 1000 Hz with a bandpass filter of 0.1–200 Hz. Electrode impedance was maintained below 10 kΩ throughout the recording. EEG signals were referenced online to the average of all electrodes, and a ground electrode was placed at the forehead. Recordings were performed in a shielded operating room to minimize electrical noise.

The EEGLAB toolbox in the MATLAB software environment was used for EEG data preprocessing, which considered both interference signals and signal integrity.

### 2.7 Frequency Band Definitions for EEG Analysis

For the computation of the weighted phase lag index (wPLI) and other spectral analyses, EEG frequency bands were defined as follows: delta (δ, 1–4 Hz), theta (θ, 4–8 Hz), alpha (α, 8–13 Hz), beta (β, 13–30 Hz), and gamma (γ, 30–45 Hz). These band ranges are consistent with standard EEG nomenclature and were applied consistently across all connectivity and power spectral analyses.

### 2.8 Functional Connectivity Analysis Using wPLI

To quantify brain functional connectivity, we computed the wPLI for each canonical frequency band (see *Frequency Band Definitions* for details) using the FieldTrip toolbox implemented in MATLAB (RRID:SCR_004849). wPLI measures the consistency of phase differences between two neuroelectric signals while minimizing the confounding effects of volume conduction and noise, thereby providing a robust estimate of true neural synchronization.

For each frequency band, wPLI was computed using a multitaper method based on Fourier transforms of 2‐s Hanning‐windowed epochs with 50% overlap. Phase synchronization was estimated only for channel pairs with significant phase‐locking values (PLV > 0.1) to reduce spurious connectivity. For each participant, wPLI was then calculated for all pairwise combinations of the 64 scalp electrodes, yielding a 64 × 64 connectivity matrix per frequency band. The resulting matrices were symmetric and were not thresholded or binarized prior to averaging. To capture global network dynamics relevant to consciousness recovery, we averaged wPLI values across all channel pairs to derive a whole‐brain connectivity index for each frequency band.

### 2.9 Efficacy Assessment

Anesthetic efficacy was evaluated by two neurologists who had no conflicts of interest with the research team certified in the use of the CRS‐R, as follows: The baseline CRS‐R evaluation was completed within 7 days preceding the intervention and served as a reference for evaluating subsequent changes in neurological function. Post‐intervention evaluations were conducted systematically at 3 months after the intervention to capture medium‐term outcomes. To minimize bias, the evaluating neurologists remained blinded to participants' group allocation throughout the trial. All assessments were performed independently, and any discrepancies in scores between the two examiners were resolved through discussion to consensus or by a third blinded evaluator if necessary. All CRS‐R scores and observational notes were recorded on a standardized electronic case report form. Audio or video recordings of the assessments were made with participants' consent to allow retrospective verification and quality control. Regular meetings were held with evaluators to ensure adherence to CRS‐R protocols and maintain rating consistency throughout the study period.

### 2.10 Statistical Analysis

Data were analyzed using SPSS software (version 22.0). Normality was assessed using the Shapiro–Wilk test. Continuous data with a normal distribution are presented as mean ± standard deviation (SD) and were compared using independent‐samples *t*‐tests. Non‐normally distributed data are reported as median (interquartile range) and were analyzed with the Mann–Whitney *U* test. Categorical variables are summarized as frequencies and percentages and were compared using Fisher's exact test. *p* < 0.05 was considered statistically significant.

To enhance comparability between the two groups and mitigate potential confounding by indication, we rigorously compared baseline demographic and clinical characteristics between the esketamine and propofol groups. As further control for potential residual confounding factors in this non‐randomized study, we performed supplementary multivariate analyses for the primary outcomes. Specifically, for the primary clinical outcome of consciousness improvement at 3 months (binary variable: improved vs. not improved), a multiple logistic regression model was fitted, adjusting for predefined potential confounders, that is, age, etiology (TBI vs. CVD), and preoperative CRS‐R score. For the time to respiratory recovery, another multiple linear regression model was applied, adjusting for the same set of covariates.

To investigate the link between intraoperative EEG measures and consciousness recovery after 3 months, we conducted exploratory logistic regression analyses. We examined the primary outcome (3‐month consciousness improvement, binary) based on anesthetic type (esketamine vs. propofol), using PE during anesthesia and gamma‐band wPLI during recovery as covariates. These EEG metrics were chosen due to significant differences between groups in the primary analysis (PE: *p* < 0.001; gamma wPLI: *p* = 0.054). The models were adjusted for confounders (age, etiology, preoperative CRS‐R score) and model fit was evaluated using likelihood ratio tests. A two‐tailed *p* < 0.05 was deemed significant for the tests, and *p* < 0.05 indicated an independent association for individual predictors.

To control for type I error inflation due to multiple comparisons in EEG analyses, the false discovery rate (FDR) was adjusted using the Benjamini–Hochberg procedure. FDR correction was applied specifically to the following comparisons between the esketamine and propofol groups for EEG‐derived metrics:

1. PE values at each time‐point across the anesthesia timeline (preoperative, anesthesia maintenance, and recovery phases).
2. wPLI values within each of the five standard frequency bands (delta, theta, alpha, beta, and gamma) during specific states (e.g., preoperative, anesthesia maintenance, and recovery phases).

For these FDR‐corrected comparisons, a corrected *p*‐value (*p*_FDR_) < 0.05 was considered statistically significant.

### 2.11 Cluster‐Based Statistical Analysis for Time‐Frequency Data

To statistically compare the time‐frequency representations (TFRs) between the esketamine and propofol groups, a non‐parametric cluster‐based permutation test was employed, as implemented in the FieldTrip toolbox. This method effectively controls for the multiple comparisons problem across time points and frequency bins. First, for each subject, TFRs were computed using a sliding time window. Second, independent‐samples *t*‐tests were performed at each time‐frequency point between groups. Third, adjacent time‐frequency points with *t*‐statistics exceeding a preset threshold (uncorrected *p* < 0.05) were grouped into clusters. The cluster‐level statistic was defined as the sum of the *t*‐statistics within each cluster. Finally, the significance of the observed clusters was evaluated by comparing them against a null distribution generated through 1000 random permutations of group labels. Clusters with a permutation‐based *p*‐value (*p*_cluster_ < 0.05) (two‐tailed) were considered statistically significant.

Effect sizes are reported (Cohen's *d* for *t*‐tests, rank‐biserial correlation [*r*] for Mann–Whitney *U* tests, and Cramér's *V* for Fisher's exact test) to quantify the magnitude of between‐group differences.

Detailed information on DoC diagnosis using the CRS‐R, perioperative hemodynamic management, anesthesia recovery process, and EEG preprocessing procedures (including large‐amplitude noise removal, frequency filtering, artifact deletion using the Artifact Subspace Reconstruction algorithm, and downsampling) is provided in the Supporting Information.

## 3 Results

### 3.1 General Data Comparisons

Baseline demographic and clinical characteristics are compared between the esketamine and propofol groups in Table 1. We found no statistically significant differences in key baseline variables, such as age, sex, etiology, disease duration, preoperative level of consciousness, and CRS‐R scores (all *p* > 0.05), indicating that the two groups were well balanced. Esketamine significantly reduced postoperative recovery time for spontaneous breathing compared to propofol (*p* < 0.05) and required less intraoperative norepinephrine for circulatory stability (*p* < 0.05).

**TABLE 1:** Comparison of the general information of the two patient groups.

	Esketamine group (*n* = 17)	Propofol group (*n* = 17)	*t‐*value	*p*
Sex				> 0.999
M	12 (70.59%)	10 (58.82%)		
F	5 (29.41%)	7 (41.18%)		
Age (years)	46.92 ± 15.18	50.29 ± 14.00	1.46	0.154
Disease duration (months)	4 (3, 6)	3 (3, 6)	−1.049	0.294
Etiology				0.481
TBI	9 (52.94%)	10 (58.82%)		0.732
CVD	8 (47.06%)	7 (41.18%)		
Level of consciousness				0.465
UWS	10 (58.80%)	13 (76.47%)		
MCS−	6 (35.29%)	4 (23.53%)		
MCS+	1 (5.91%)	0 (0%)		
CRS‐R score	7.65 ± 1.84	6.47 ± 2.10	−1.742	0.091
Surgical time (min)	51.92 ± 10.9	54.17 ± 6.28	−0.403	0.691
Time for respiratory recovery (min)	12.02 ± 3.88	17.42 ± 4.62	−3.108	0.005
Norepinephrine				0.034
Yes	3 (17.65%)	9 (52.94%)		
No	14 (82.35%)	8 (47.06%)		

*Note:* Data are presented as mean ± SD, median (IQR), or *n* (%). Between‐group comparisons were performed using the independent‐samples *t*‐test (reported with Cohen's *d*), Mann–Whitney *U* test (reported with rank‐biserial correlation *r*), or Fisher's exact test (reported with Cramér's *V*).

Abbreviations: CRS‐R, Coma Recovery Scale–Revised; CVD, cerebrovascular disease; F, female; M, male; MCS, minimally conscious state; TBI, traumatic brain injury; UWS, unresponsive wakefulness syndrome.

### 3.2 Comparison of Electroencephalographic Time‐Frequency Spectra and Power Spectra for Two Drugs (A: Propofol, B: Esketamine) in DoC

**FIGURE 2:**
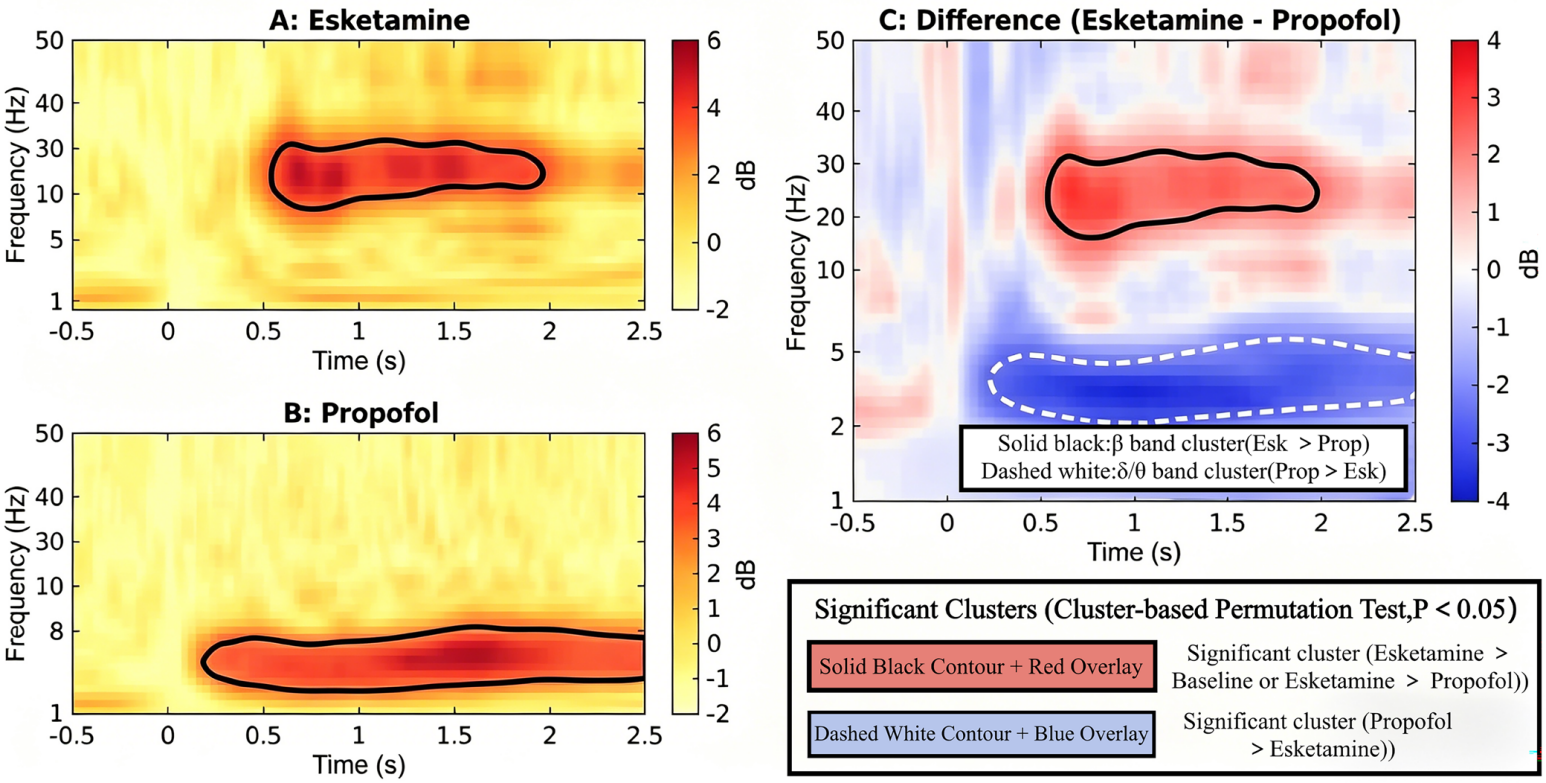
Time‐frequency analysis of EEG power and significant clusters identified by cluster‐based permutation tests. (A) Time‐frequency representation of the Esketamine group. (B) Time‐frequency representation of the Propofol group. Color bars indicate power density (dB). (C) Difference plot (Esketamine minus Propofol). Significant clusters are highlighted with contour lines based on non‐parametric cluster‐based permutation tests (*p* < 0.05). Solid black contours indicate regions where Esketamine power is significantly higher than Propofol (mainly in the β band, 15–30 Hz). Dashed white contours indicate regions where Propofol power is significantly higher than Esketamine (mainly in the δ band, 1–4 Hz).

The time‐frequency spectrum analysis (Figure 2A,B) revealed distinct patterns of oscillatory power modulation between groups. To rigorously quantify these differences, cluster‐based permutation tests were performed. As illustrated in the difference plot (Figure 2C), two statistically significant clusters were identified (*p*_cluster_ < 0.05). First, a significant positive cluster (indicated by solid black contours in Figure 2A,C) was observed in the beta band (15–30 Hz), demonstrating that esketamine maintained significantly higher high‐frequency activity compared to propofol during the maintenance phase. Second, a significant negative cluster (indicated by dashed white contours in Figure 2B,C) was found in the delta/theta band (1–8 Hz), reflecting that propofol induced more pronounced low‐frequency slowing. These statistical results confirm that esketamine preserves high‐frequency neural complexity, whereas propofol is characterized by dominant slow‐wave oscillations.

### 3.3 Impact of Propofol and Esketamine on Brain Electrical Complexity During Various Anesthesia Stages

**FIGURE 3:**
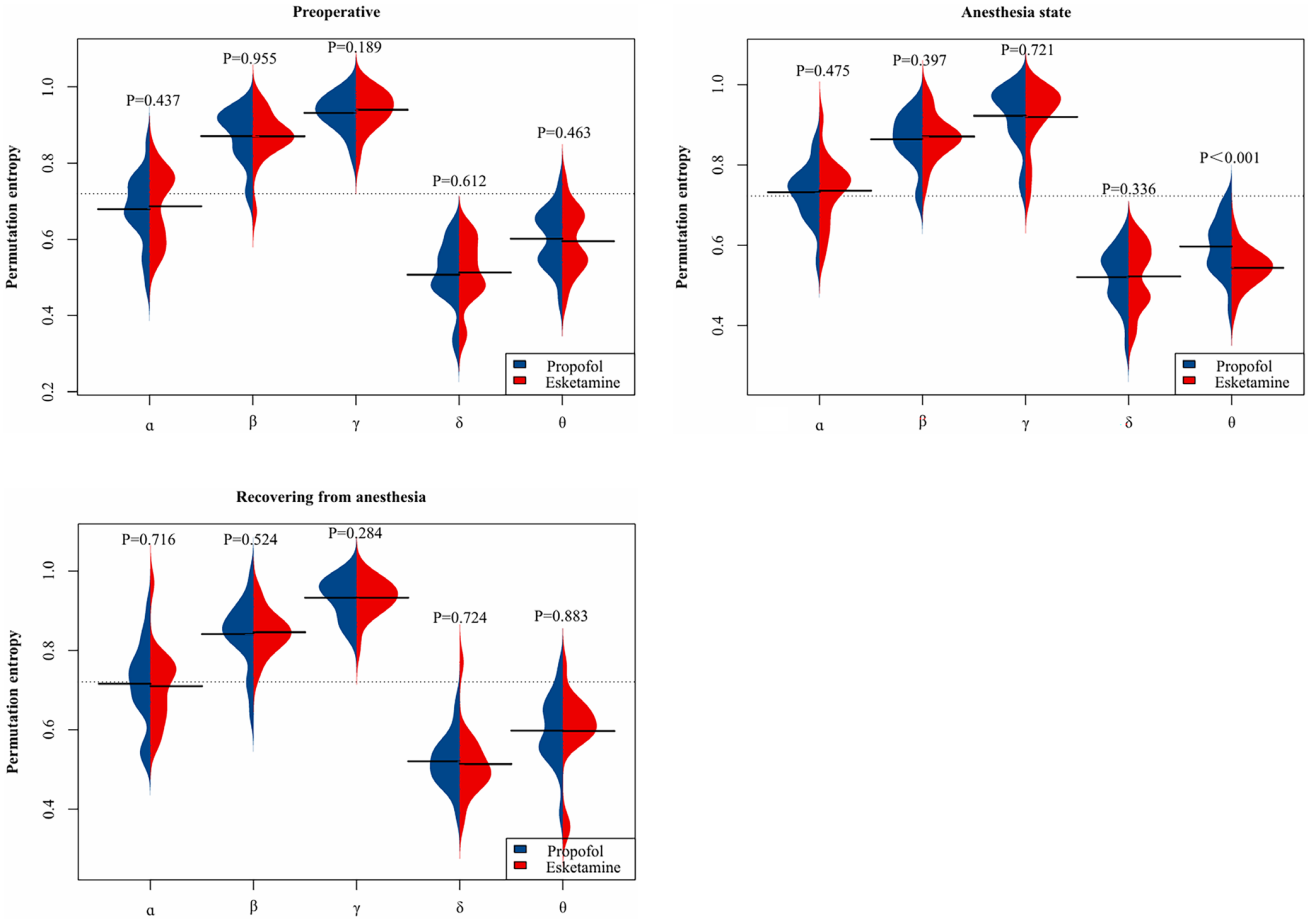
Comparison of the effects of propofol and esketamine on brain electrical complexity at different anesthesia stages. *Preoperative*: Comparing PE between the propofol and esketamine groups shows no significant difference in baseline electroencephalography complexity, as indicated by box plots (key time‐point: *p* = 0.955). *Anesthesia Maintenance*: During anesthesia maintenance, the esketamine group showed significantly higher PE values than the propofol group (*p* < 0.001), with entropy values falling to the 0–0.8 range, indicating greater suppression of brain electrical complexity. No significant differences were found between the groups at other times (*p* = 0.336). *Anesthesia Recovery*: Analysis of PE progression during the recovery phase revealed no significant differences between the groups at any time‐point (*p* = 0.724). Furthermore, the entropy values reverted to the range of 0.2–1.0, approximating preoperative levels. PE, permutation entropy.

During anesthesia maintenance, the PE values of the esketamine group were significantly higher than those of the propofol group (*p*_FDR_ < 0.001), indicating that esketamine preserved a higher cerebral electrical complexity than did propofol. At all other time‐points during this phase, no statistically significant differences were observed (*p* = 0.336), highlighting the predominant influence of anesthetic depth. During the recovery phase, entropy values in both groups returned to preoperative levels (0.2–1.0), and no significant differences were detected between the groups at any time‐point (typical *p* = 0.724), confirming that restoration of neural complexity during recovery is independent of the type of anesthetic administered (Figure 3).

### 3.4 Evaluation of Brain Functional Connectivity Modifications Throughout Preoperative, Anesthesia, and Recovery Intervals

Anesthesia has been demonstrated to suppress whole‐brain functional connectivity markedly, as indicated by a general reduction in wPLI values across all frequency bands (all < 0.25), with the high‐frequency gamma band showing the most pronounced suppression (decreasing from 0.5 in the awake state to 0.1 during anesthesia). In contrast, low‐frequency bands such as delta/theta are relatively unaffected (ca. 0.25), resulting in a low‐frequency‐dominant pattern.

**FIGURE 4:**
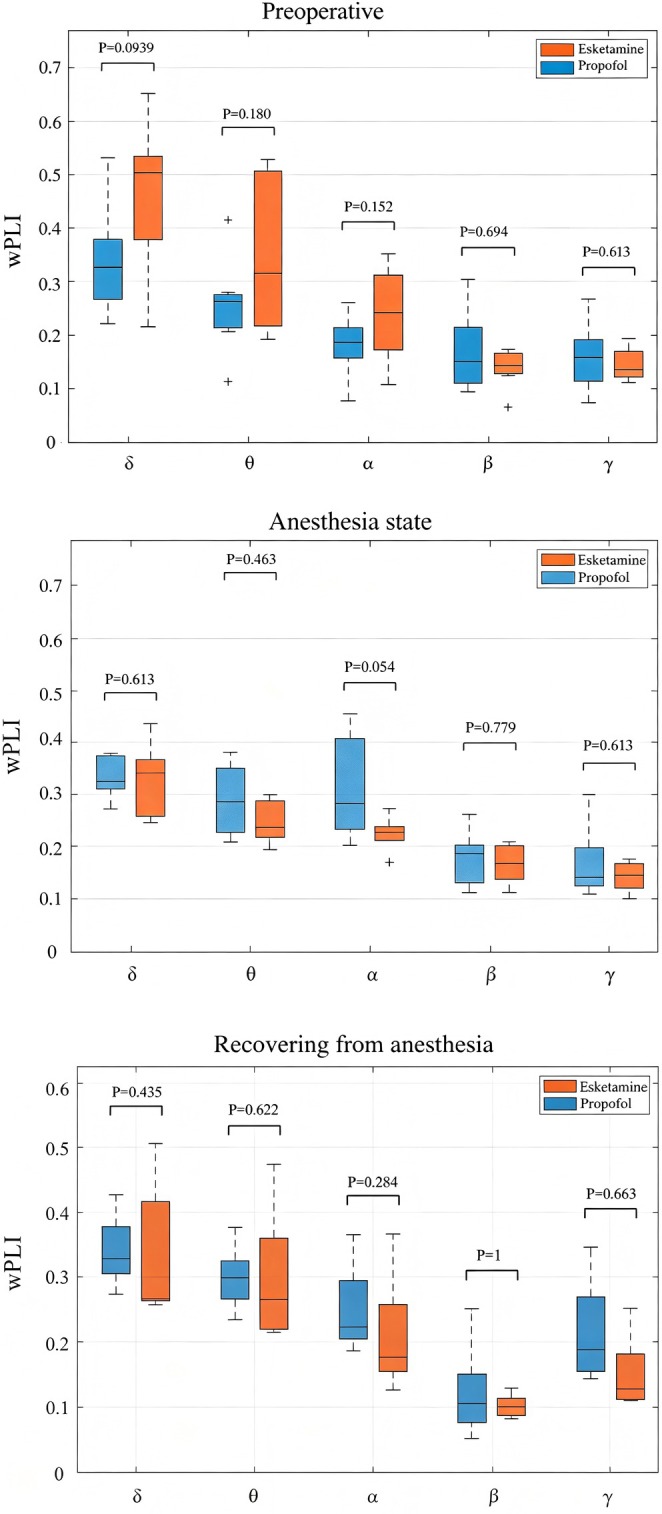
Comparison of functional electroencephalography connectivity in different states. *Preoperative state*: The high‐frequency gamma band is predominant (wPLI ca. 0.5), with the intensity hierarchy of the bands being γ > β > α > θ > δ, indicating activation of physiological high‐level cognitive networks. Significant fluctuations were observed in the δ/θ band (*p* = 0.094–0.189), reflecting normal dynamic brain regulation. *Anesthesia maintenance*: Suppression is seen across all frequency bands (wPLI < 0.25), with pronounced suppression of the γ band (0.1) and a relative preservation of low‐frequency δ/θ bands (ca. 0.25), resulting in a low‐frequency‐dominant pattern with δ/θ > γ/β. The high stability of wPLI values (*p* > 0.5 in 9/10 cases) indicates a uniform blockade of functional connectivity. *Recovery state*: The δ band rebounded to its peak (0.35), while γ band recovery was delayed (0.25), leading to an inversion in band strength: δ > θ > α > γ > β. Significant changes at the edge of the γ band (*p* = 0.065) suggests that reorganization of the higher cognitive network was incomplete, indicating an incomplete return of consciousness. wPLI, weighted phase lag index.

During the recovery phase, an atypical recovery trajectory was observed: the delta band rebounded first, to the highest level (approximately 0.35), whereas the gamma band showed a delayed recovery (reaching only 0.25). This led to an inverted frequency band strength order (delta > theta > alpha > gamma > beta), which starkly contrasted with the high‐frequency dominant pattern observed in the awake state (gamma > beta > alpha > theta > delta) (Figure 4).

### 3.5 Differential Effects of Propofol and Esketamine on Brain Network Connectivity in Different Time Periods

Propofol showed a strong trend toward attenuating high‐frequency connectivity in the gamma band (*p*_FDR_ = 0.054), whereas esketamine induced a rebound in theta‐band connectivity that approached, but did not reach, statistical significance (*p*_FDR_ = 0.065). Despite the lack of strict FDR correction, the large effect sizes and the consistency of these frequency‐specific patterns with the known mechanisms of each drug are suggestive of the biological relevance of these findings. The effect of esketamine is attributed to the preservation of high‐frequency activity potential, which expedites the transition from a state of reduced consciousness to full wakefulness. Conversely, propofol, which is characterized by its potent gamma inhibition, results in delayed neurological recovery, as evidenced by a non‐significant improvement in the theta band (*p* = 0.435) and a significant delay in synchrony within the alpha band (8–12 Hz) (*p* = 0.622). This delayed recovery ultimately extends the awakening process (Figure 4).

### 3.6 Comparison of Consciousness Recovery Between Propofol and Esketamine at 3‐Months Postoperatively

**FIGURE 5:**
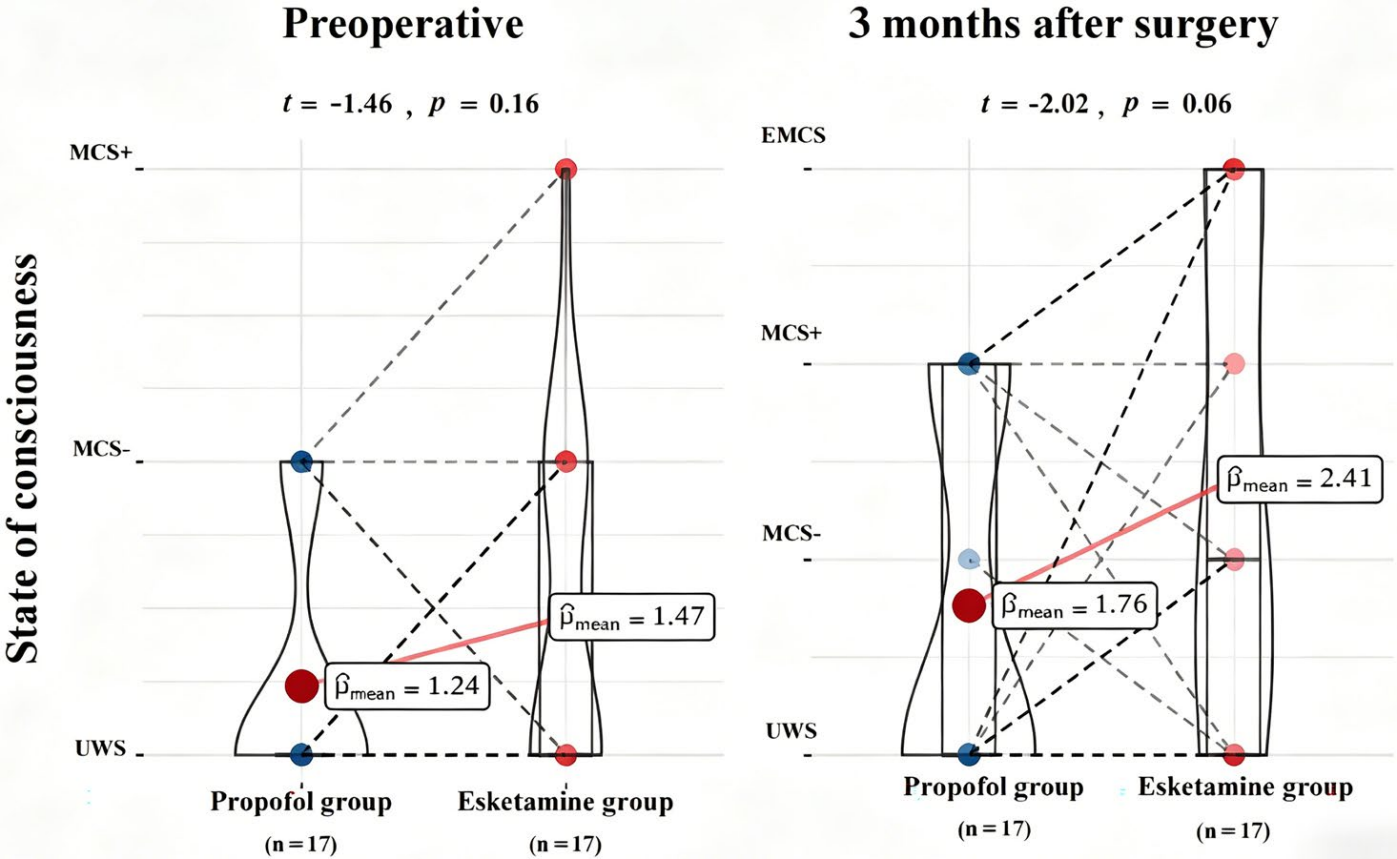
Comparison of consciousness recovery between the esketamine and propofol groups before and at 3 months after surgery. Patient states were classified as UWS, MCS−, MCS+, and EMCS. Pmean values indicate the average score for each group at these time‐points. *T*‐tests were used for statistical analysis: Preoperative *t* = −1.46, *p* = 0.16; 3‐month follow‐up *t* = −2.02, *p* = 0.06. *p* = 0.06 suggests a non‐significant trend toward better recovery with esketamine at 3 months. EMCS, emergence from minimally conscious state; MCS, minimally conscious state; UWS, unresponsive wakefulness syndrome.

Recovery of consciousness at 3‐months postsurgery was compared between patients administered esketamine or propofol. Preoperatively, no significant difference in consciousness levels was observed between the groups (*t* = −1.46, *p* = 0.16). At the 3‐month follow‐up, although the difference did not reach statistical significance, a strong trend toward a higher level of consciousness recovery was noted in the esketamine group than in the propofol group (*t* = −2.02, *p* = 0.06). This trend was further supported by the higher mean values observed across patient categories (MCS+, MCS−, emergence from minimally conscious state (EMCS), UWS) in the esketamine group, suggesting a potential beneficial effect of esketamine on long‐term consciousness recovery (Figure 5).

### 3.7 Multivariable Analysis of Primary Outcomes

To address potential confounding factors inherent in our non‐randomized study design, we conducted multivariable analyses of the primary outcomes. Utilizing a multiple logistic regression model, we adjusted for age, etiology (traumatic brain injury [TBI] versus cerebrovascular disease [CVD]), and preoperative CRS‐R scores. The analysis revealed that the esketamine group was significantly associated with increased odds of consciousness improvement at three months (adjusted Odds Ratio [aOR] = 6.84, 95% Confidence Interval [CI]: 1.42–32.98, *p* = 0.017). Similarly, a multiple linear regression model, adjusted for the same covariates, demonstrated that esketamine was independently associated with a reduced time to respiratory recovery (adjusted *β* coefficient = −5.21 min, 95% CI: −8.63 to −1.79, *p* = 0.004) (Table 2).

**TABLE 2:** Multivariable analysis of primary outcomes adjusting for potential confounders.

Variable	Adjusted odds ratio (aOR)/*β* coefficient^a^	95% confidence interval	*p*
Consciousness improvement at 3 months (logistic regression)			
Anesthetic: Esketamine (vs. Propofol)	6.84	1.42 to 32.98	0.017
Age	0.99	0.94 to 1.04	0.642
Etiology: TBI (vs. CVD)	1.32	0.38 to 4.61	0.662
Preoperative CRS‐R score	1.28	0.91 to 1.80	0.156
Time to respiratory recovery (minutes) (linear regression)			
Anesthetic: Esketamine (vs. Propofol)	−5.21^a^	−8.63 to −1.79	0.004
Age	0.07	−0.08 to 0.22	0.347
Etiology: TBI (vs. CVD)	−1.05	−3.91 to 1.81	0.463
Preoperative CRS‐R score	−0.28	−0.89 to 0.33	0.361

Abbreviations: aOR, adjusted Odds Ratio; CI, Confidence Interval; CRS‐R, Coma Recovery Scale–Revised; CVD, cerebrovascular disease; TBI, traumatic brain injury.

^a^ *β* Coefficient from multiple linear regression model.

### 3.8 Exploratory Analysis of EEG Measures and Consciousness Recovery

To assess whether intraoperative EEG metrics contributed to the observed differences in 3‐month consciousness recovery, we performed an exploratory logistic regression analysis. In a model adjusting for age, etiology, and preoperative CRS‐R score, we included permutation entropy (PE) during anesthesia maintenance and gamma‐band weighted phase lag index (wPLI) during recovery—both of which showed significant or near‐significant between‐group differences in primary analyses. Esketamine remained independently associated with higher odds of consciousness improvement (adjusted odds ratio [aOR] = 5.21; 95% CI: 1.10–24.7; *p* = 0.038), although the effect estimate was attenuated compared to the model without EEG covariates (aOR = 6.84). Gamma‐band wPLI was independently associated with improved recovery (aOR per 0.1 increase = 2.10; 95% CI: 1.12–3.94; *p* = 0.021), while PE showed a trend toward significance (aOR per 0.1 increase = 1.45; 95% CI: 0.98–2.15; *p* = 0.062). Inclusion of both EEG covariates significantly improved model fit (likelihood ratio *χ* ^2^ = 6.82; *p* = 0.033).

## 4 Discussion

In this study, we compared the effectiveness of propofol and esketamine for anesthesia in patients with DoC. Esketamine yielded a notably reduced time to spontaneous breathing recovery and more stable intraoperative hemodynamics. At the 3‐month follow‐up, esketamine resulted in significantly more improvements in consciousness levels than did propofol. This enhanced long‐term recovery involved the maintenance of neural network complexity and functional connectivity under esketamine anesthesia, as indicated by significantly higher PE and wPLI.

We demonstrated that anesthesia maintenance with propofol significantly decreased whole‐brain PE [26], particularly within the theta, alpha, and high‐frequency bands. This may indicate a decline in the randomness and unpredictability of brain signals, consistent with the known broad suppression of cortical information processing of propofol, which involves enhanced GABAA receptor‐mediated inhibitory neurotransmission [27, 28, 29]. This results in a high degree of synchronization, order, and “rigidity” in neural network activity [30, 31]. This state contrasts with the characteristic “criticality” of the conscious brain, a highly complex, optimal information processing condition that balances order and disorder [32, 33, 34]. This effect was accompanied by a generalized reduction in whole‐brain functional connectivity, as measured by the wPLI, further corroborating the profound and widespread propofol‐induced inhibition and decoupling of thalamocortical and corticocortical information flow [35, 36, 37, 38]. This extensive inhibition was particularly pronounced in the diminished connectivity within the high‐frequency gamma band, which is associated with cognitive integration. This disrupts information exchange between functional brain modules, resulting in a functionally “fragmented” brain [39, 40], which is fundamentally detrimental to global dynamic information integration that is necessary for consciousness. The disruption's extent, especially the suppression of gamma‐band connectivity, may directly impact recovery potential, as indicated by our exploratory analyses connecting intraoperative EEG metrics to outcomes at 3 months.

Although esketamine also decreased PE during anesthesia, the decrease magnitude was significantly smaller than that observed in the propofol group, and PE returned to baseline levels rapidly during the recovery period. Its reduced wPLI inhibition and relatively better connectivity preservation in higher‐frequency bands (e.g., gamma) were particularly noteworthy. This neurophysiological profile agreed with the known NMDA receptor antagonism mechanism of esketamine, which, unlike GABAergic agent such as propofol, does not typically cause widespread suppression of neural firing [40]. Instead, esketamine may induce a state of “connected consciousness” or pharmacologic dissociation, potentially through cortical circuit disinhibition [41, 42, 43, 44, 45]. Preservation of a higher degree of functional network architecture during unconsciousness may facilitate a more rapid and organized return to consciousness.

Esketamine significantly reduced the postoperative recovery time for spontaneous respiration by 31% as compared to propofol (12.02 ± 3.88 min vs. 17.42 ± 4.62 min, *p* = 0.005). Additionally, the use of intraoperative vasoactive medications to maintain circulatory stability was substantially lower in the esketamine than propofol group (17.65% vs. 52.94%, respectively). EEG analysis revealed a significant rebound increase in PE within the delta frequency band (1–4 Hz) after esketamine anesthesia. Although delta oscillations are traditionally perceived as indicating brain inhibition or dormancy [46, 47], emerging evidence suggests a close association with cortical–subcortical network activation and enhanced sympathetic tone in specific contexts [48, 49]. The observed triad of increased delta entropy, accelerated respiratory recovery, and enhanced circulatory stability suggests that esketamine exerts a disinhibitory effect on key brainstem structures via NMDA antagonism. This could, in turn, activate autonomic and respiratory regulators, such as the paraventricular nucleus of the thalamus and the nucleus of the solitary tract within the brainstem, thereby significantly enhancing sympathetic output, as indicated by the stabilization or elevation of heart rate and blood pressure [50, 51, 52]. Additionally, modulation of brainstem respiratory centers, particularly the rostral ventrolateral medulla, may contribute to maintaining respiratory drive and may counteract the respiratory‐depressant effects of opioids, such as remifentanil which was co‐administered in this study [53, 54].

In contrast, propofol exhibits bidirectional inhibitory effects. It not only extensively suppresses complex activity in the cerebral cortex by enhancing GABAergic inhibition, as demonstrated by a reduction in full‐frequency power, but also significantly inhibits activity in the cardiovascular and respiratory centers of the brainstem. This widespread autonomic nervous system suppression is directly linked to a high incidence of intraoperative hypotension, necessitating the increased use of antihypertensive medications and delaying postoperative respiratory center activation. Consequently, the clinical advantages of esketamine, which are fundamentally attributed to its distinctive neurophysiological mechanisms at the systemic level, provide a compelling justification for its application in neurosurgical anesthesia, where autonomic function preservation is critical.

We also observed that esketamine facilitated long‐term consciousness recovery. At 3‐month follow‐up, the esketamine group showed significantly greater improvement in consciousness levels compared to propofol (*p* = 0.012). Exploratory analyses provided insight into the potential mechanisms underlying this benefit: when gamma‐band wPLI during recovery and PE during maintenance were included as covariates in the regression model, the effect size of esketamine on 3‐month recovery was attenuated (aOR reduced from 6.84 to 5.21), and both EEG metrics contributed independently to the outcome (gamma wPLI: aOR per 0.1 increase = 2.10, *p* = 0.021; PE: aOR per 0.1 increase = 1.45, *p* = 0.062). Inclusion of these metrics significantly improved model fit (likelihood ratio *p* = 0.033), suggesting that preservation of neural complexity and high‐frequency connectivity may partially mediate the relationship between anesthetic choice and long‐term consciousness recovery. These neurophysiological effects align with known mechanisms of esketamine. While our study did not measure molecular markers, esketamine was previously shown to promote neuroplasticity via Brain‐derived neurotrophic factor/mammalian target of rapamycin pathway(BDNF/mTOR) [55, 56, 57]. Such mechanisms may plausibly have contributed to the enhanced recovery observed in our patients. This preservation of information integration capacity provides the necessary “computational basis” for consciousness restoration [58, 59]. The near‐complete recovery of beta‐band wPLI during the recovery phase facilitates efficient thalamocortical information flow reestablishment, which is directly associated with the global network connectivity essential for clarity of consciousness and the EMCS state [60, 61, 62, 63]. The etiological heterogeneity mechanism, as revealed by time‐frequency analysis, indicates that acepromazine consistently enhances delta power inhibition in patients with CVD to prevent excitotoxicity and reestablish the corticothalamic loop [43, 44, 64, 65]. Conversely, activation of the hippocampal–prefrontal BDNF/mTOR pathway promotes neuroplastic repair through dynamic theta band fluctuations in patients with TBI [66, 67]. This etiology‐specific neuromodulatory property provides insights for the debates regarding drug efficacy in hypoxic encephalopathy [68, 69], and affirming that etiology is a critical determinant of drug response.

Although our study demonstrated significant improvement in consciousness recovery at the 3‐month follow‐up in the esketamine group, it is important to acknowledge that this time frame may not be sufficient to fully capture the long‐term neuroprotective or neuromodulatory effects of esketamine. While the preservation of EEG complexity and functional connectivity, along with accelerated respiratory and hemodynamic recovery, suggests a favorable neurophysiological profile, true neuroprotection implies sustained structural and functional benefits beyond the acute recovery phase. Future studies with extended follow‐up periods (e.g., 6–12 months) are warranted to determine whether these early advantages translate into lasting improvements in functional independence, cognitive performance, and quality of life. Additionally, incorporating multimodal neuroimaging (e.g., fMRI, PET) and biomarker assessments (e.g., BDNF, inflammatory cytokines) could elucidate the molecular and circuit‐level mechanisms underlying esketamine's potential neurorestorative effects.

## 5 Limitations

This study had some limitations. Despite the non‐randomized design, the groups were well balanced at baseline, and our primary findings were robust to sensitivity analyses adjusted for key potential confounders. However, the modest sample size limited the statistical power for more extensive subgroup analyses (e.g., based on etiology) and the generalizability of our findings. The 3‐month follow‐up may have been insufficient to determine the long‐term sustainability of the observed cognitive benefits. Additionally, although all patients received remifentanil, we cannot entirely rule out its potential confounding effects on EEG dynamics and recovery metrics. Finally, the lack of a placebo control group meant that we could not definitively exclude the influence of the surgical procedure itself or that of natural recovery trajectories. However, in this pilot study, we intended to assess the feasibility and safety of the protocol, generate initial hypotheses, and obtain compelling effect size estimates to inform the design of a future definitive, large‐scale, multicenter trial. Despite the abovementioned limitations, the large effect sizes and multimodal concordance of our data provide a robust foundation for future large‐scale multicenter trials.

## 6 Conclusions

This study found that, compared with propofol, esketamine better maintained EEG complexity, preserved high‐frequency connectivity, reduced respiratory recovery time, and stabilized circulation in patients with DoC. At 3 months postoperatively, esketamine was superior to propofol in the rate of consciousness improvement, supporting its role in facilitating neurorecovery in patients with DoC. However, the 3‐month follow‐up period limits our ability to definitively conclude long‐term neuroprotective efficacy. Future large‐scale, randomized controlled trials with longer follow‐up durations and integrative neurobiological assessments are needed to validate these preliminary findings and to elucidate the underlying neuromodulatory mechanisms.

## Author Contributions

X.Q., X.C., H.N., and B.W. collected the clinical and EEG data, whereas X.Q., X.C., and H.N. performed postoperative follow‐ups and evaluations. Data analysis was performed by X.Q., X.C., Z.Z., and J.W. using software. X.Q. and X.C. drafted the manuscript, which was then reviewed by L.Y., X.G., J.H., Z.L., and X.L. X.Q., X.C., J.W., and Z.Z. conducted statistical analyses. L.Y. and X.L. supervized this study. All authors have read and approved the final manuscript.

## Funding

This study was financially supported by the Ministry of Science and Technology of the People's Republic of China grant STI2030‐Major Projects+2021ZD0204300.

## Ethics Statement

Ethical clearance was provided by the institutional review committee at Peking University International Hospital, and consent was secured from all patients (Approval No. 2024‐KY‐0062‐02).

## Conflicts of Interest

The authors declare no conflicts of interest.

## Supporting information


**Data S1:** The Supporting Informations include detailed criteria and scoring protocols for Doc diagnosis using the CRS‐R. It also provides comprehensive clinical protocols for perioperative hemodynamic management and anesthesia recovery. Furthermore, it details the in‐depth methodologies for high‐density EEG data preprocessing (including large‐amplitude noise removal, frequency filtering, artifact deletion using the Artifact Subspace Reconstruction algorithm, and downsampling), along with the justification and post hoc power analysis for the study's sample size.

## Data Availability

The data that support the findings of this study are available from the corresponding author upon reasonable request.
