# Supplementary material for: Esketamine Preserves Network Connectivity and Promotes Recovery in Consciousness Disorders
Source: CNS Neurosci Ther. 2026 May 13;32(5):e70890. doi: 10.1002/cns.70890 (PMC13170474; doi:10.1002/cns.70890)
Supplement: Supplementary file 1 — Data S1: The Supporting Informations include detailed criteria and scoring protocols for Doc diagnosis using the CRS‐R. It also provides comprehensive clinical protocols for perioperative hemodynamic management and anesthesia recovery. Furthermore, it details the in‐depth methodologies for high‐density EEG data preprocessing (including large‐amplitude noise removal, frequency filtering, artifact deletion using the Artifact Subspace Reconstruction algorithm, and downsampling), along with the justification and post hoc power analysis for the study's sample size. [file CNS-32-e70890-s001.docx]

**Supplementary Materials**

**DoC Diagnosis**

UWS and MCS were diagnosed using the CRS-R. This scale evaluates six neurobehavioral dimensions, i.e., auditory, visual, motor, speech, communication, and arousal functions, using 23 standardized test items. Each assessment was conducted following a predetermined protocol, with scores based on the CRS-R manual and ranging from 0 to 2 points per item. The overall score was calculated as the sum of scores across all dimensions.

**Perioperative Management and Anesthesia Recovery Process**

Throughout surgery, an individualized hemodynamic management protocol was implemented. Intravenous ephedrine (3–6 mg) was administered when the systolic blood pressure decreased > 20% from baseline. Continuous infusion of norepinephrine was initiated if required. The acid–base balance and electrolyte concentrations were meticulously maintained within physiological limits, as verified by real-time blood gas analysis.

Anesthetic drug infusion was discontinued immediately postoperatively and patients were transferred to the post-anesthesia care unit. Sugammadex sodium was intravenously infused to reverse neuromuscular-blocking effects. Recovery during spontaneous breathing was continuously monitored. When SpO₂ recovered to preoperative baseline values ± 3% in a deoxygenated state, and hemodynamic parameters stabilized within normal ranges, patients were transferred to the general ward.

**Removal of Large-Amplitude Noise**

The collected data were imported into the EEGLAB toolbox. Each patient's data were marked out using the eegplot.m function with a time-window of 100 ms and an amplitude of 200 μV. Loop noise is likely to occur at points of abrupt data change. Large-amplitude noise, defined as signals exceeding ±200 μV, based on established EEG preprocessing protocols for patients with DoC, also appear at the marked points in the EEG signal on the BIS monitor. These large noises were first visually identified in a 5-s time-window and then manually excluded after independent cross-verification by two trained EEG analysts, to ensure reliability.

**Removal of Low- and High-Frequency EEG Components and Power Frequency Interference**

The pop_eegfiltnew.m function (EEGLAB toolbox) was used to remove the 0.1-Hz low-frequency component of the EEG signal. A window-function finite impulse response (FIR) filter or two-way least-squares method was used to reduce baseline drift in EEG data. A linear-phase FIR filter was used to remove high-frequency signals (> 45 Hz).

Electromagnetic interference of 50 Hz and its narrow vicinity, caused by background AC power, was reduced by using an adaptive notch filter.

**Artifact Deletion**

The Artifact Subspace Reconstruction (ASR) algorithm was applied to remove artifactual data, using a standard deviation cutoff threshold of 20 (consistent with the EEGLAB default for clinical data) and a window length of 0.5 s. The Clean_rawdata function of EEGLAB was used to remove residual artifactual portions that were not fully corrected by the ASR, by adjusting the window length to 1 s, as previously recommended.^1^

**Reducing Computational Pressure**

Because some data time series were longer, the EEG signal was downsampled from 128 Hz to 100 Hz to effectively reduce the computational pressure, while ensuring EEG signal integrity. Data were downsampled to 64 Hz to reduce the computational load, while maintaining the Nyquist frequency (32 Hz) well above the highest frequency of interest (30 Hz after filtering), thus robustly preventing aliasing.^2^

**Sample Size Justification**

A formal *a priori* sample size calculation was not performed due to the exploratory nature of this mechanistic investigation and the lack of prior effect size estimates for our primary outcomes in the DoC population.

A sample size of 34 patients (17 per group) was determined primarily based on practical feasibility (patient recruitment rate, study duration, and resource constraints), and was consistent with sample sizes commonly employed in previous pilot and mechanistic studies in patients with DoC.^3^

To assess the sensitivity of our study quantitatively, a post-hoc power analysis was conducted for the primary clinical outcome, i.e., the difference in the proportion of patients with consciousness improvement at 3 months. The observed effect size corresponded to a statistical power of approximately 82%, using a two-tailed Fisher’s exact test with an alpha level of 0.05. Thus, at 17 participants per group, our study was adequately powered (i.e., >80%) to detect a large between-group difference in clinical improvement rates.

**References for Supplementary Materials**

[1] Bigdely-Shamlo N, Mullen T, Kothe C, et al. The PREP pipeline: standardized preprocessing for large-scale EEG analysis. Front Neuroinform.2015;9:16. doi:10.3389/fninf.2015.00016

[2] Reisinger P, Larochelle J, Abkai C, et al. The impact of EEG preprocessing parameters on ultra-low-power seizure detection. Epilepsia. doi:10.1111/epi.18521

[3] Duclos C, Maschke C, Mahdid Y, et al. Brain Responses to Propofol in Advance of Recovery from Coma and Disorders of Consciousness: A Preliminary Study. Am J Respir Crit Care Med. 2022;205(2):171-182. doi:10.1164/rccm.202105-1223OC
